# Expressions of CD23, IL-17 and MMP-9 in Patients with Colorectal Cancer

**Published:** 2020-02

**Authors:** Xueguang GUO, Gang LIU, Xiaoping XIE, Jing LI, Zehui HOU, Yanhong GU, Lijiang YU

**Affiliations:** 1. Department of Medical Oncology, Chinese PLA General Hospital, Beijing, China; 2. Department of Hepatobiliary & Enteric Surgery, Xiangya Hospital, Central South University, Changsha, China; 3. Department of Gastroenterology Surgery, The First Affiliated Hospital of Nanchang University, Nanchang, China; 4. Department of Oncology, Zhongshan Hospital Affiliated to Guangzhou University of Chinese Medicine, Zhongshan, China; 5. Department of Gastrointestinal and Hernia Surgery, The Sixth Affiliated Hospital of Sun Yat-sen University, Guangzhou, China; 6. Department of Oncology, The First Affiliated Hospital of Nanjing Medical University, Nanjing, China; 7. Department of Internal Medicine-Oncology, Jingjiang People’s Hospital, Jingjiang, China

**Keywords:** Clinicopathology, Colorectal cancer, Interleukin-17

## Abstract

**Background:**

We aimed to detect IL-17, MMP-9 and CD23 in serum of patients with colorectal cancer to provide some proper references for diagnosis and treatment of this disease.

**Methods:**

Overall, 287 patients with colorectal cancer were collected in the Digestive Surgery Department of Chinese PLA General Hospital, Beijing, China from January 2017 to November 2018 and were used as the study group, meanwhile, 200 people who took physical examination in the same period were used as the control group. They were retrospectively analyzed. The concentrations of IL-17, MMP-9 and CD23 in serum were detected by ELISA 10 d before and after treatment and 30 d after treatment. The relationship between IL-17, MMP-9 and CD23 concentration and clinicopathology was analyzed.

**Results:**

The concentrations of CD23, IL-17 and MMP-9 in peripheral blood of the patients in the study group were significantly higher than those in the control group (*P*<0.001). IL-17, MMP-9 and CD23 were negatively correlated with treatment time and pathological features in the study group (*P*<0.001).

**Conclusion:**

The concentrations of IL-17, MMP-9 and CD23 obviously increased in peripheral blood of patients with colorectal cancer, the three were negatively correlated with treatment time and were significantly correlated with TNM staging and differentiation degree of colorectal cancer. It is expected to estimate the illness.

## Introduction

Colorectal cancer, a malignant tumor originated from mucosal epithelium of large intestine, is one of the most common malignant tumors in digestive tract (1, 2). As one of the common tumors in clinic, colorectal cancer has an extremely high morbidity and mortality (3). There were about 1.4 million new cases in 2012, among which there were 700,000 deaths (4). At present, the treatment method of colorectal cancer is mainly surgery or chemoradiotherapy (5), but colorectal cancer has no obvious indications in early period, so it is easily ignored by patients. Once the patient is diagnosed, colorectal cancer has already been in advanced period, during this time, cancer cells have generally spread and metastasized, which makes it difficult to treat colorectal cancer by resection (6, 7) and is one of the reasons why the prognosis of colorectal cancer is poor.

The pathogenesis of colorectal cancer is not yet clear. The main function of matrix metalloproteinase-9 (MMP-9) is degrading and remodeling the dynamic balance of extracellular matrix (8). At present, MMP-9 has been abnormally expressed in tumors and participate in metastasis and invasion of tumors (9, 10). IL-17 has obvious changes in treatment of rectal cancer, but the specific mechanism has not been clear (11, 12). CD23 is a low-affinity IgE receptor expressed in dendritic cells, monocytes and B cells (13). Some scholars believe that CD23 can be produced in nasopharyngeal cancer tissue infected by EBV, the main mechanism is that the nuclear antigen of EBV nuclear antigen II, which is produced during EBV incubation period, is a necessity of B lymphocyte’s transformation, during which c-myc gene is regulated and CD23 is produced (14).

At present, the functions of IL-17, MMP-9 and CD23 in colorectal cancer have not been confirmed. However, IL-17, MMP-9 and CD23 in serum of patients with colorectal cancer were detected by experiments in this paper to provide some proper references for diagnosis and treatment of colorectal cancer in clinic in the future.

## Methods

### General data

overall, 287 patients with colorectal cancer, who were admitted to the Digestive Surgery Department of the First Affiliated Hospital of Nanjing Medical University, Beijing, China from January 2017 to November 2018, were collected and were used as the study objects. Meanwhile, 200 people who took physical examination were used as the control group, they were retrospectively analyzed.

This experiment was approved by the Ethics Committee of The First Affiliated Hospital of Nanjing Medical University, all the objects signed the informed consent form.

### Inclusion criteria and exclusion criteria

Inclusion criteria: patients conformed to clinical symptoms of colorectal cancer (15); patients were diagnosed with colorectal cancer by the pathology biopsy of The First Affiliated Hospital of Nanjing Medical University; patients received follow-up treatment in the First Affiliated Hospital of Nanjing Medical Universityl after they were diagnosed; patients had complete case data; patients were willing to cooperate with the medical workers of the hospital; patients were from 30 to 70 yr old.

Exclusion criteria: patients had other tumors, cardiovascular and cerebrovascular diseases, organ failure, liver dysfunction and kidney dysfunction, mental illness, physical disability, history of familial genetic diseases; patients could-n’t take care of themselves; patients were bedridden for long time; patients had drug allergy; patients transferred to other hospital halfway. JK-(a)-5931, the detection was carried out strictly in accordance with the instructions of the kit. The other part of the serum was used to detect CD23, Ficoll density gradient centrifugation was used to separate peripheral blood mononuclear cells (PBMC), then 5 mL of lymphocyte separation solution (Shanghai Yuanmu Biotechnology Co., Ltd., item number: YS-6131) was added into the centrifuge tube, next, anticoagulated venous blood and sterile PBS were added into it, the ratio was 1:1, lastly, the mixture was mixed equably. A pipette was used to add liquid drop-wise until to the layered liquid level, then the mixture was centrifuged horizontally at 400 g×30 min. The upper liquid was discarded, then the pipette was inserted into cloud layer to collect monocytes and transferred them into another tube. PBS with a volume of 5 times was added into the tube, then the mixture was centrifuged at 300 g×10 min, the cells were rinsed twice, the supernatant was discarded, next, red blood cell lysate was added into the mixture, it was incubated for 2 minutes at room temperature, next, PBS was added into the mixture, the mixture was rinsed twice. Finally, the supernatant was discarded, RPMI-1640 medium with 10% of PBS (Wuhan Chundu Biotechnology Co., Ltd., item number: CDLG-5404) was added into the mixture. The resuspended cells were counted by Cellqutst software.

### Observation indicators

The concentrations of IL-17, MMP-9 and CD23 in serum of the patients in the study group were detected 10 d before and after treatment and 30 d after treatment. The correlation among IL-17, MMP-9, CD23 and treatment time was analyzed. The relationship between the concentrations of IL-17, MMP-9, CD23 in the study group and clinicopathology was analyzed.

### Statistical methods

All the experiment results were calculated by SPSS24.0 statistical software (Shanghai Yuchuang Network Technology Co., Ltd.). All the graphs were drawn by Graphpad8 software (Shenzhen Softhead Technology Co., Ltd.) and the results were checked twice. The measurement data were expressed in the form of rate, chi-square test was used in the comparison between groups. The enumeration data were expressed in the form of mean value ± standard deviation, t-test was used in the comparison between groups. Variance analysis of repeated measure was used in the comparison in groups at different time points. Correlation analysis was performed by Spearman correlation analysis. *P*<0.050 was considered to be statistically significant.

## Results

### The comparison of the general data

The general data of the patients in two groups were compared, including age, BMI, platelet, red blood cell, white blood cell count, gender, nationality, residence, marriage, smoking, drinking, exercise habits (Table 1).

**Table 1:** The comparison of the clinical data of the patients in two groups [n (%)]

***Variable***	***The study group (n=287)***	***The control group (n=200)***	***t/2***	***P***
Age (yr)	47.82±10.54	46.33±9.84	1.577	0.116
BMI (kG/m^2^)	22.84±4.2	22.17±5.04	1.592	0.112
Platelet (×10^9^cells/L)	228.21±50.14	220.42±57.21	1.591	0.112
Red blood cell (×10^12^cells/L)	4.66±0.64	4.72±0.51	1.104	0.270
White blood cell (×10^9^cells/L)	8.15±1.17	7.96±1.34	1.660	0.098
Gender: Male	184 (64.11)	142 (71.00)	2.527	0.112
Female	103 (35.89)	58 (29.00)		
Nationality: Han nationality	282 (98.26)	198 (99.00)	0.458	0.498
Minority	5 (1.74)	2 (1.00)		
Residence: City	235 (81.88)	172 (86.00)	1.456	0.228
Countryside	52 (18.12)	28 (14.00)		
Marriage: Married	261 (90.94)	175 (87.50)	1.488	0.223
Unmarried	26 (9.06)	25 (12.50)		
Smoking: Yes	197 (68.64)	150 (75.00)		
No	90 (31.36)	50 (25.00)		
Drinking: Yes	212 (73.87)	142 (71.00)	0.488	0.485
No	75 (26.13)	58 (29.00)		
Exercise: Yes	31 (10.80)	26 (13.00)	0.551	0.458
No	256 (89.20)	174 (87.00)		
TNM staging^*^: I∼II	168 (58.54)			
III∼IV	119 (41.46)			
Differentiation degree				
Middle differentiation and low differentiation	157 (54.70)			
High differentiation	130 (45.30)			
Lymph node metastasis				
Yes	108 (37.63)			
No	179 (62.37)			
Infiltration degree				
Infiltrating to serosa	139 (48.43)			
Not infiltrating to serosa	148 (51.57)			
The type of tumor				
Villous adenoma	125 (43.55)			
Villous adenoma	125 (43.55)			

Note: TNM staging criteria refer to AJCC cancer staging manual (14)

**Fig. 1:**
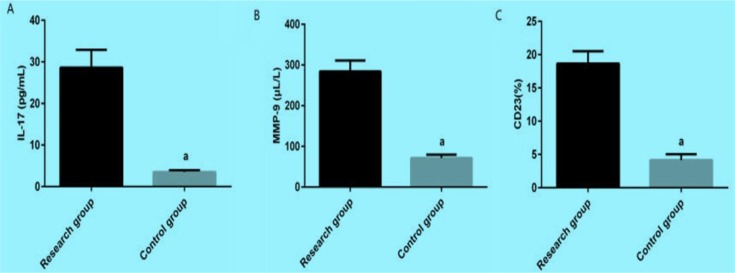
The comparison of the concentrations of IL-17, MMP-9 and CD23 between two groups. A: The comparison of the concentrations of IL-17 between two groups. After detected by ELISA, the concentration of IL-17 in the study group is significantly higher than that in the control group. a means compared with the concentration of IL-17 in the study group, *P*<0.001. B: The comparison of the concentrations of MMP-9 between two groups. After detected by ELISA, the concentration of MMP-9 in the study group is significantly higher than that in the control group. a means compared with the concentration of MMP-9 in the study group, *P*<0.001. C: The comparison of the concentrations of CD23 between two groups. After detected by ELISA, the concentration of CD23 in the study group is significantly higher than that in the control group. a means compared with the concentration of CD23 in the study group, *P*<0.001

The concentrations of IL-17, MMP-9 and CD23 in serum of the study group before treatment were significantly higher than those in the control group, *P*<0.001 (Fig. 1).

### The changes of IL-17, MMP-9 and CD23 in the study group before and after treatment

**Fig. 2:**
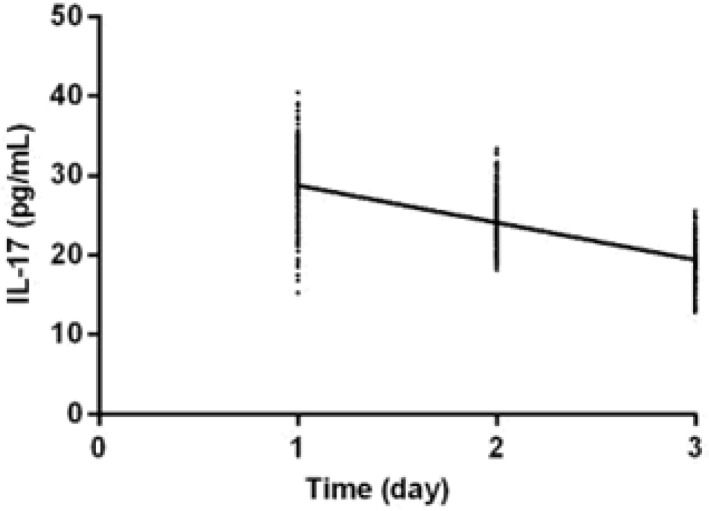
The correlation analysis between IL-17 and treatment time. IL-17 was negatively correlated with treatment time in the study group (r=−0.757, *P*<0.001)

**Fig. 3:**
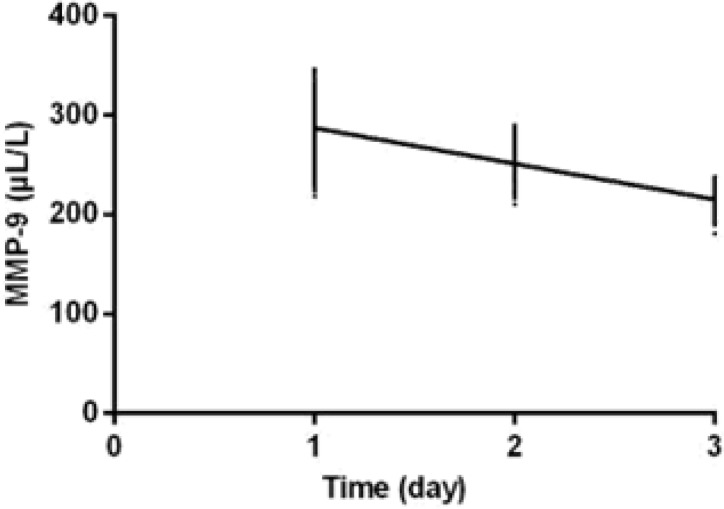
The correlation analysis between MMP-9 and treatment time. MMP-9 was negatively correlated with treatment time in the study group (r=−0.847, *P*<0.001)

**Fig. 4:**
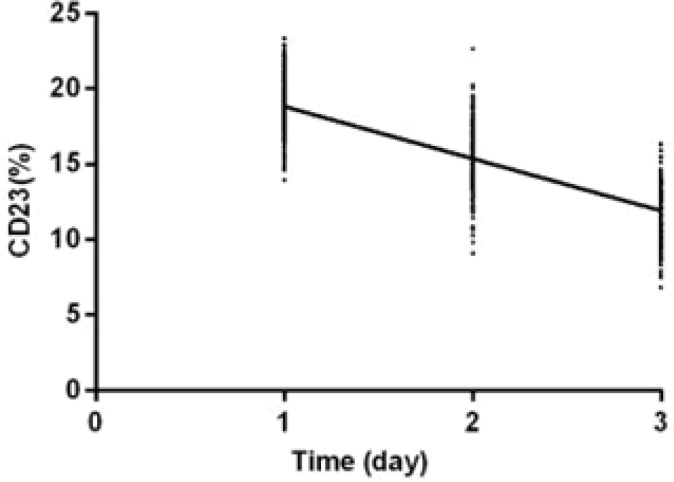
The correlation analysis between CD23 and treatment time. CD23 was negatively correlated with treatment time in the study group (r=−0.851, *P*<0.001)

The levels of IL-17, MMP-9 and CD23 in the study group 10 d after treatment were significantly lower than those before treatment (*P*<0.001). The levels of IL-17, MMP-9 and CD23 30 d after treatment were significantly lower than those 10 d after treatment (*P*<0.001). IL-17, MMP-9, and CD23 were negatively correlated with the treatment time of the study group (r=−0.757, −0.847, −0.851, *P*<0.001) (Table 2, Fig. 2–4).

**Table 2:** The changes of IL-17, MMP-9 and CD23 in the study group before and after treatment

***Variable***	***IL-17 (pg/m)***	***MMP-9 (μL/)***	***CD23 (%)***
Before treatment	28.63±4.26	284.62±26.21	18.66±1.86
10 d after treatment	24.66±3.05^a^	256.72±15.05^a^	15.57±2.06^a^
30 d after treatment	19.15±2.42^ab^	212.83±10.52^ab^	11.94±1.57^ab^
F	2982.107	7172.243	2950.184
*P*	< 0.001	< 0.001	< 0.001

Note: a means compared with IL-17, MMP-9, CD23 in the same group before treatment, *P*<0.001; b means compared with IL-17, MMP-9, CD23 in the same group 10 d after treatment, *P*<0.001

### The relationship between the concentrations of IL-17, MMP-9, CD23 in the study group and clinicopathology

IL-17, MMP-9 and CD23 was related to TNM staging, differentiation degree, lymph node metastasis, and infiltration degree (*P*<0.001) (Table 3–5).

**Table 3:** The relationship between the concentration of IL-17 and clinicopathology in the study group (pg/mL)

***Variable***	***n***	***Concentration***	**t**	**P**
Age (yr) > 60	164	27.94±4.56	0.328	0.743
≤60	123	28.11±4.05		
BMI (KG/m^2^): > 26	189	28.04±4.15	0.205	0.838
≤26	98	28.15±4.62		
Gender : Male	184	28.36±4.04	0.820	0.413
Female	103	27.95±4.11		
Nationality : Han nationality	282	27.86±4.12	0.108	0.915
Minority	5	28.06±4.32		
Residence : City	235	28.11±4.15	0.251	0.802
Countryside	52	27.95±4.25		
Marriage: Married	261	28.65±4.62	0.467	0.641
Unmarried	26	28.21±4.22		
Smoking :Yes	197	27.86±4.15	0.247	0.805
No	90	28.07±4.03		
Drinking :Yes	212	28.16±4.03	0.365	0.716
No	75	28.36±4.22		
Exercise : Yes	31	27.68±4.62	0.112	0.911
No	256	27.58±4.72		
TNM staging : I∼II	168	24.51±3.62	11.312	< 0.001
III∼IV	119	30.17±4.86		
Differentiation degree			13.783	< 0.001
Middle differentiation and low differentiation	157	31.87±2.57		
High differentiation	130	26.63±3.84		
Lymph node metastasis : Yes	108	31.52±3.84	11.142	< 0.001
No	179	25.66±4.58		
Infiltration degree			17.582	< 0.001
Infiltrating to serosa	139	31.84±2.66		
Not infiltrating to serosa	148	24.83±3.59		
The type of tumor			0.147	0.883
Villous adenoma	125	28.24±4.15		
Adenoma	162	28.17±3.86		

**Table 4:** The relationship between the concentration of MMP-9 and clinicopathology in the study group (μL/L)

Variable		***n***	***Concentration***	***t***	***P***
Age (yr)	> 60	164	280.17±28.11	0.409	0.683
	≤60	123	281.51±26.57		
BMI (KG/m^2^)	> 26	189	287.15±25.16	0.884	0.378
	≤26	98	284.51±21.56		
Gender	Male	184	279.56±26.15	1.286	0.200
	Female	103	275.46±25.48		
Nationality	Han nationality	282	289.56±29.15	0.308	0.759
	Minority	5	285.51±30.45		
Residence	City	235	284.11±30.84	0.444	0.657
	Countryside	52	286.15±25.64		
Marriage	Married	261	281.51±25.26	1.462	0.145
	Unmarried	26	289.14±26.51		
Smoking	Yes	197	287.15±26.48	0.593	0.553
	No	90	289.15±26.52		
Drinking	Yes	212	294.85±48.56	0.544	0.587
	No	75	298.45±51.15		
Exercise	Yes	31	281.06±25.61	1.759	0.080
	No	256	289.84±26.32		
TNM staging	I∼II	168	248.41±35.51	13.982	< 0.001
	III∼IV	119	308.51±36.41		
Differentiation degree	Middle differentiation and low differentiation	157	311.51±26.56	12.501	< 0.001
	High differentiation	130	265.16±36.15		
Lymph node metastasis	Yes	108	298.86±28.52	10.468	< 0.001
	No	179	260.85±30.55		
Infiltration degree	Infiltrating to serosa	139	278.15±35.44	0.733	0.464
	Not infiltrating to serosa	148	280.85±26.55		
The type of tumor	Villous adenoma	125	287.41±27.56	0.231	0.818
	Adenoma	162	288.15±26.51		

**Table 5:** The relationship between the concentration of CD23 and clinicopathology in the study group (%)

***Variable***		***n***	***Concentration***	***t***	***P***
Age (yr)	> 60	164	17.89±1.85	0.723	0.470
	≤60	123	18.04±1.58		
BMI (KG/m^2^)	> 26	189	18.44±1.46	1.525	0.128
	≤26	98	18.15±1.65		
Gender	Male	184	17.68±1.15	1.134	0.258
	Female	103	17.84±1.14		
Nationality	Han nationality	282	18.16±1.15	0.019	0.985
	Minority	5	18.15±1.84		
Residence	City	235	17.15±1.89	1.095	0.275
	Countryside	52	17.46±1.64		
Marriage	Married	261	18.07±1.52	0.659	0.511
	Unmarried	26	17.86±1.84		
Smoking	Yes	197	18.48±1.68	1.628	0.105
	No	90	18.84±1.86		
Drinking	Yes	212	17.48±1.16	1.086	0.278
	No	75	17.65±1.18		
Exercise	Yes	31	17.75±1.81	1.235	0.218
	No	256	18.15±1.69		
TNM staging	I∼II	168	16.89±1.54	13.912	< 0.001
	III∼IV	119	19.66±1.82		
Differentiation degree	Middle differentiation and low differentiation	157	20.59±2.89	12.763	< 0.001
	High differentiation	130	17.15±1.15		
Lymph node metastasis	Yes	108	18.56±1.15	1.910	0.057
	No	179	18.27±1.30		
Infiltration degree	Infiltrating to serosa	139	21.55±2.56	20.733	< 0.001
	Not infiltrating to serosa	148	16.15±1.81		
The type of tumor	Villous adenoma	125	18.65±1.15	1.138	0.256
	Adenoma	162	18.48±1.33		

## Discussion

In recent years, with the change of dietary habits, the incidence of various digestive tract tumors has been increasing (16–18). Many factors and genes may be involved in the occurrence and development of colorectal cancer (19–22), but the functions of IL-17, MMP-9 and CD23 in colorectal cancer have not yet been clear. ROP-γt ubiquitination inhibits the occurrence of colonic inflammation mediated by IL-17 and tumors (23). As an inactivated form of 10 kDa propeptide in non-small cell lung cancer, MMP-9 can facilitate secretion of cells (24).

Due to the biological function of MMP-9, like cutting gelatin, it can be activated by other MMP or tissue plasminogen activator (tPA)-plasmin system, also, it can facilitate the movement of malignant cells. CD23 is involved in the occurrence and developmentof small cell lymphoma (23–25). This study analyzed the correlation between the three and colorectal cancer as well as the relationship between the three and clinicopathology to verify the functions of IL-17, MMP-9 and CD23 in colorectal cancer.

The results of this experiment showed that the concentrations of IL-17, MMP-9 and CD23 in peripheral blood of patients with colorectal cancer were significantly increased, suggesting that IL-17, MMP-9 and CD23 might be involved in occurrence and development of colorectal cancer. IL-17, MMP-9 and CD23 were negatively correlated with treatment time, suggesting that the recovery of patients with colorectal cancer can be estimated by detecting the concentrations of IL-17, MMP-9 and CD23 in peripheral blood of patients. IL-17, MMP-9 and CD23 were obviously related to TNM staging and differentiation degree of colorectal cancer, suggesting that the severity of colorectal cancer can be estimated by detecting the concentrations of IL-17, MMP-9 and CD23 in peripheral blood of patients in clinic.

In this study, the concentration of IL-17 obviously increased before the patients with colorectal cancer were treated, but it obviously decreased after treatment. This result is consistent with other studies (26–28), where the concentration of IL-17 also obviously increased and it was related to susceptibility of gastric cancer, which could support the results of this experiment.

It is speculated that the mechanism of IL-17, which involves in development of colorectal cancer, is inhibiting proliferation and activation of NK cells, lymphocytes’ ability to secrete cytokines, and proliferation of T cells and facilitating angiogenesis of tumors, as well as inducing metastasis and infiltration of the nidus of colorectal cancer through cytokines (29). MMP-9 is a zinc ion proteolytic enzyme that can trigger a variety of inflammations (30), suggesting that the mechanism that MMP-9 involves in colorectal cancer is also associated with inflammation. CD23 can facilitate synthesis and secretion of IgE, mediate adhesion between cells, and help basophils to release histamine. CD23 facilitates synthesis of IgE when the concentration of IgE decreases and inhibits synthesis of IgE when the concentration of IgE increases, which is often used as an activation marker of B cells (31). The concentration of CD23 increases in serum of patients with colorectal cancer, indicating dysfunction of B cells, imbalance of cell subsets, and destruction of microbial environment in patients with colorectal cancer.

There are some limitations in this study. For example, the mechanism of IL-17, MMP-9 and CD23 in colorectal cancer needs to be further investigated to verify our results. At present, there are few studies about the functions of IL-17, MMP-9 and CD23 in colorectal cancer at home and abroad, so it is hard to cite a number of other experiment results to carry out comparison and discussion. The difference between the results of this experiment and the results of other experiments cannot be excluded. Moreover, the experiment period was short, so it was difficult to estimate the effects of IL-17, MMP-9 and CD23 on prognosis of colorectal cancer.

## Conclusion

The concentrations of IL-17, MMP-9 and CD23 obviously increase in peripheral blood of patients with colorectal cancer, they are negatively correlated with treatment time and are significantly correlated with TNM staging and differentiation degree of colorectal cancer. It is expected to estimate illness progression and treatment of patients with colorectal cancer by detecting IL-17, MMP-9 and CD23 in the future.

## Ethical considerations

Ethical issues (Including plagiarism, informed consent, misconduct, data fabrication and/or falsification, double publication and/or submission, redundancy, etc.) have been completely observed by the authors.
